# Emerging Nanotechnology for Treatment of Alzheimer’s and Parkinson’s Disease

**DOI:** 10.3389/fbioe.2021.672594

**Published:** 2021-05-25

**Authors:** Amanda Li, Joel Tyson, Shivni Patel, Meer Patel, Sruthi Katakam, Xiaobo Mao, Weiwei He

**Affiliations:** ^1^Washington University School of Medicine, St. Louis, MO, United States; ^2^Department of Chemical, Biochemical and Environmental Engineering, University of Maryland Baltimore County, Baltimore, MD, United States; ^3^Neuroregeneration and Stem Cell Programs, Institute for Cell Engineering, Johns Hopkins University School of Medicine, Baltimore, MD, United States; ^4^Department of Neurology, Johns Hopkins University School of Medicine, Baltimore, MD, United States; ^5^Key Laboratory of Micro-Nano Materials for Energy Storage and Conversion of Henan Province, Henan Joint International Research Laboratory of Nanomaterials for Energy and Catalysis, College of Chemical and Materials Engineering, Institute of Surface Micro and Nano Materials, Xuchang University, Xuchang, China

**Keywords:** Parkinson’s disease, Alzheimer’s disease, nanotechnology/nanomaterials, oxidative stress, nanozymes

## Abstract

The prevalence of the two most common neurodegenerative diseases, Parkinson’s disease (PD) and Alzheimer’s Disease (AD), are expected to rise alongside the progressive aging of society. Both PD and AD are classified as proteinopathies with misfolded proteins α-synuclein, amyloid-β, and tau. Emerging evidence suggests that these misfolded aggregates are prion-like proteins that induce pathological cell-to-cell spreading, which is a major driver in pathogenesis. Additional factors that can further affect pathology spreading include oxidative stress, mitochondrial damage, inflammation, and cell death. Nanomaterials present advantages over traditional chemical or biological therapeutic approaches at targeting these specific mechanisms. They can have intrinsic properties that lead to a decrease in oxidative stress or an ability to bind and disaggregate fibrils. Additionally, nanomaterials enhance transportation across the blood-brain barrier, are easily functionalized, increase drug half-lives, protect cargo from immune detection, and provide a physical structure that can support cell growth. This review highlights emergent nanomaterials with these advantages that target oxidative stress, the fibrillization process, inflammation, and aid in regenerative medicine for both PD and AD.

## Introduction

Protein aggregation is a typical histopathological hallmark in Parkinson’s disease (PD) and Alzheimer’s Disease (AD). PD is characterized by the aggregation of misfolded α-synuclein (α-syn) protein in inclusions called Lewy bodies (LB) in dopaminergic neurons, resulting in severe motor dysfunction. Alzheimer’s Disease is characterized by abnormal accumulation of amyloid-β (Aβ) plaque and tau neurofibrillary tangles, resulting in brain damage affecting critical cognitive processes. Emerging clinical and experimental results support the hypothesis that pathological α-syn, Aβ, and tau are prion-like peptides/proteins that can induce the propagation of endogenous monomers, and cause proteinopathy spreading from cell-to-cell (Braak et al., 2003; Kordower et al., 2008; Li et al., 2008; Luk et al., 2012; Guo et al., 2016; Mao et al., 2016; Tyson et al., 2016; He et al., 2018; Kam et al., 2018; Kim et al., 2019).

Native α-syn undergoes a misfolding process from soluble and random conformation to the insoluble and fibrillar form in pathological conditions. When misfolded α-syn aggregates, it localizes in the mitochondria, inducing mitochondrial fragmentation and decreased membrane potential (Li et al., 2007; Devi et al., 2008; Ganjam et al., 2019). The aggregation of Aβ peptide is thought to be a result of dysfunctional mitochondrial reactive oxygen species (ROS) production and dyshomeostasis of metals from oxidative stress (Tönnies and Trushina, 2017; Gupta et al., 2019; Poulson et al., 2019). In AD, microtubule-associated tau protein is known to undergo abnormal hyperphosphorylation, leading to tau tangles with prion-like activity. Though the mechanism and effects of tau tangles are not yet well understood, there is some promise that tau is an effective therapeutic target (Binder et al., 2005; Gong and Iqbal, 2008; Iqbal et al., 2016; Takeda, 2019).

Similar pathological mechanisms in both diseases cause enhanced production of ROS leading to a cascade of oxidative stress (Li et al., 2007; Devi et al., 2008; Goedert, 2015; Tönnies and Trushina, 2017; Hassanzadeh and Rahimmi, 2018; Ganjam et al., 2019; Gupta et al., 2019). With increased cell stress, microglial reactions, and increased expression of inflammatory cytokines, both diseases significantly increase neuronal inflammation, which is thought to promote cell death and further protein/peptide aggregation (Tufekci et al., 2012; Lema Tomé et al., 2013; Rivest, 2015; Gupta et al., 2019). The biological mechanisms that affect both PD and AD as described are misfolded protein aggregation, oxidative stress, inflammation, and cell death (de Bem et al., 2021).

Despite decades of clinical trials using traditional therapeutics, highly successful treatment of both oxidative stress and protein misfolding in neurodegenerative diseases has been elusive (Querfurth and LaFerla, 2010). Combating amyloidosis in both AD and PD with small molecules, peptides, and monoclonal antibodies especially, has resulted in little success. This leaves the door open for nanomaterials with appealing physicochemical properties, tenability, and multifunctionality to improve understanding and treatment of the diseases (Andrikopoulos et al., 2020; Chen P. et al., 2020; Ke et al., 2020; Pichla et al., 2020; Kakinen et al., 2021).

Nanotechnologies are increasingly being used in biomedical applications, and more treatments for neurodegenerative disorders are expected to emerge. Nanomaterial formulations have shown the ability to alleviate oxidative stress and inflammation directly (Wang et al., 2019; Eleftheriadou et al., 2020), and to overcome barriers in passage across the blood brain barrier (BBB) (Ulbrich and Lamprecht, 2010; Leyva-Gómez et al., 2015), although the specific mechanism for delivery across the BBB varies and has not yet been fully elucidated. Traditional therapeutic drugs are also likely to have off-target effects. Nanomaterials demonstrate the ability to improve localized targeted delivery of disease therapeutics by enhancing the dosing efficacy of delivered drugs, controlling cargo release profiles, and by being functionalized for the specific biological target of interest (Su and Kang, 2020). Additionally, composite nanomaterials are being developed to improve regenerative medicine techniques and encourage new cell growth in PD and AD (Bordoni et al., 2020).

Nanoparticles (NPs) can enhance the transport of therapeutics across the BBB during pathological conditions in PD and AD. Characteristics of disease-afflicted BBB include greater vascular permeability, decreased expression of tight junctions and BBB transporters, and the build-up of blood-derived debris and cells into perivascular spaces (Sweeney et al., 2018; Huang et al., 2020). Such pathological conditions impair concentration gradient-driven diffusion, decreasing the function of carrier-mediated transport (CMT) and receptor-mediated transport (RMT) (Sweeney et al., 2018). These conditions pose additional concerns due to the increased chance of agents getting trapped in the enlarged perivascular spaces (Wang et al., 2018). Although CMT has been a difficult transport route for NP systems due to the high selectivity of carrier proteins (Curley and Cady, 2018), RMT has been found to be more conducive to NP systems and is currently the most common type of transport for NP entry into the brain (Saraiva et al., 2016). To use RMT for transport into the brain parenchyma for NP systems, NPs can be coated with ligands (such as insulin, transferrin, lactoferrin) or surfactants (such as polysorbate 80) capable of undergoing RMT (Saraiva et al., 2016; Lopalco et al., 2018). Nanoparticles functionalized by cationic substances such as albumin can cross the BBB by adsorptive transcytosis (AMT) (Lu et al., 2012), or travel across the BBB via cell-mediated transcytosis, a form of transport that relies on immune cell phagocytosis of the NPs (Chen and Liu, 2012).

In this mini-review article, we will explore the current field of nanomaterials for therapeutic application in PD and AD and highlight emerging trends and materials that appear to be forging a path toward a multifaceted approach to the similar pathologies of the diseases. The present review is divided into major sections describing important therapeutic trends: addressing oxidative stress and mitochondrial damage, prevention of α-syn, Aβ, and tau aggregation and cell-to-cell spreading, addressing inflammation, and aiding cellular regeneration (Table 1).

**TABLE 1:** Nanotechnology addressing mechanisms of PD and AD.

Biological target	Therapeutic approach		References
Oxidative stress	ROS scavengers	Antioxidant mimicking metal oxide NPs and other nanozymes, enhancing oxidative stress tolerance	Singh et al., 2017; Liu et al., 2020; Ma et al., 2020; Ruotolo et al., 2020; Yu et al., 2020
	Antioxidant drug delivery	Antioxidant liposomes (resveratrol, baicalein, curcumin, EGCG), lipoic acid capped gold, tunable hydrogels, catalase exosomes	Wang et al., 2011; Elnaggar et al., 2015; Haney et al., 2015; Ethemoglu et al., 2017; Rajput et al., 2018; Huang et al., 2019; Lai et al., 2019; Adnet et al., 2020; Chen W. et al., 2020; Piersimoni et al., 2020; Kuo et al., 2021
	Nanoemulsions	Intranasal delivery of vitamin E, coenzyme Q10 through nasal mucosa	Gupta et al., 2018; Gaba et al., 2019
Protein Aggregation	Protein degradation	Graphene quantum dots, gold NPs, light sensitive nanoassemblies	Lee et al., 2018; Tanimoto et al., 2012; Gao et al., 2016, 2019; Zhang et al., 2016; Chung et al., 2017; Kim et al., 2018; Javed et al., 2019; Zhang H. et al., 2020
	Fibrillization inhibition	Metal oxide NPs, solid lipid NPs, cerium oxide NPs, siRANA exosomes, hydrogels	Hossain and Mukherjee, 2013; Cooper et al., 2014; Jiang et al., 2016, 2018; Vakilinezhad et al., 2018; Yau and Tycko, 2018; Ma et al., 2020; Mahapatra et al., 2020; Ruotolo et al., 2020; Simpson et al., 2020
Inflammation	Lipoic acid capped gold NPs, ibuprofen nanoemulsion, loaded NPs, mitochondria targeting nanozymes		Testa et al., 2014; Mandal et al., 2016; Ganesan et al., 2019; Piersimoni et al., 2020; Zhang L. et al., 2020
Regenerative Medicine	Neutrophic factor particles and hydrogel scaffolds, self-assembling peptides		Yu et al., 2014; Cui et al., 2016; Adil et al., 2017; Moriarty et al., 2017; Struzyna et al., 2018; Moriarty et al., 2019; Bordoni et al., 2020

## Nanomaterial Approaches to Relieving Oxidative Stress in PD and AD

### Direct Antioxidant Nanoparticles

This section focuses on metal nanomaterials that directly relieve oxidative stress by serving as ROS and nitric oxide (NO) scavengers, mimicking the major antioxidant enzymes involved in oxidative-stress response including metal oxides, and redox nanozymes that mimic redox enzymes catalase, superoxide dismutase (SOD), and other antioxidants (Ambani et al., 1975; Riederer et al., 1989; Abraham et al., 2005; Li et al., 2020).

Nanozymes are nanomaterials with enzyme-like properties, tunable catalytic activity, high stability, and often the ability to simultaneously mimic multiple enzymes (Liang and Yan, 2019). Reaction mechanisms of nanozymes may vary based on their specific composition. Non-metallic nanozymes, such as carbon-based nanozymes, contain an aromatic ring that facilitates electron transfer, imitating the function of the porphyrin ring present in natural enzymes (Gao et al., 2020). Metal oxide nanozymes contain metal sites that imitate the metal catalytic active site of natural metalloenzymes. Metal oxide nanozymes tend to exhibit peroxidase-like activity, catalyzing the oxidation of a chromogenic substrate in the presence of hydrogen peroxide (Gao et al., 2020). Certain metal oxide nanoparticle systems such as cerium oxide can mimic multiple enzymes at once, and display a mechanism resembling that of redox enzymes due to their ability to switch between oxidation states (Yang et al., 2016; Hegazy et al., 2017). Metal-based nanozymes can be optimized by forming bimetallic nanoparticles, such as the PtCu system discussed below. Bimetallic nanozymes can mimic single or multiple enzymes simultaneously, and the catalytic activity of a bimetallic nanozyme system can be controlled by adjusting the ratio of the metals (He et al., 2017; Liu et al., 2020).

Cellular PD models demonstrate the potential of nanomaterials to reduce oxidative stress. A study by Ruotolo et al. examined the effects of cerium oxide NPs (CeO_2_ NPs) on a yeast cell model overexpressing human α-syn. Results showed that CeO_2_ NPs significantly reduced α-syn cytotoxicity in a dose-dependent manner through inhibition of α-syn cytoplasmic inclusion formation, and counteracted α-syn-induced mitochondrial damage. Upon treatment with CeO_2_ NPs, α-syn-expressing yeast cells possessed lower levels of mitochondrial fragmentation, considerably higher amounts of actively functioning mitochondria, and a significantly smaller pool of free radicals (Ruotolo et al., 2020). Hao et al. (2019) demonstrated the ability of copper-based NPs (particularly Cu_2_O and CuO) to eliminate ROS in a neuronal cell model of PD induced by 1-methy-4-phenylpyridinium (MPP+). Hao continues the study with mice, inducing PD with 1-methyl-4-phenyl-1,2,3,6-tetrahydropyridine (MPTP). The study results showed that Cu_*x*_O nanoclusters mimic the activity of peroxidase, superoxide dismutase, catalase, and glutathione peroxidase, thus inhibiting neurotoxicity (Hao et al., 2019). In another cellular study using a PD model induced by MPP+, Singh et al. (2017) found that Mn_3_O_4_ nanozymes effectively mimic SOD, catalase, and glutathione peroxidase, which are three major antioxidant enzymes with normally cytoprotective roles that are hampered in PD. The ability of Mn_3_O_4_ nanozymes to simultaneously mimic all three major antioxidant enzymes is significant, as each antioxidant enzyme serves a different role in combating oxidative stress. Moreover, simultaneous expression of all three major antioxidant enzymes has been found to enhance tolerance oxidative stress in plant-based models (Lee et al., 2007; Sharma et al., 2012). Liu et al. (2020) injected preformed fibrils (PFF) of α-syn to create a sporadic PD model in neuronal cells and mice. PtCu bimetallic nanoalloys (NAs) were formulated and their antioxidant capacity was quantified using standard radical 2,2-diphenyl-1-picrylhydrazyl (DPPH). Liu et al. (2021) demonstrated that PtCu NAs display peroxidase, catalase, and SOD-like activity, and can scavenge DPPH, making them promising antioxidants. Results of their study showed that the PtCu nanozyme is significantly efficient at preventing prion-like α-syn spreading in PD (Liu et al., 2020; Figure 1E). The study demonstrated by proof of concept that redox nanozymes can be promising therapeutic strategies against the pathological spread of α-syn. Further study into the optimization of nanozymes against prion-like propagation would be worthwhile, as nanozyme therapy may serve as an effective strategy against PD and other prion-like proteinopathies.

In recent studies with transgenic mice, metal oxides are used as antioxidant foundations of nanozymes, functionalized with Aβ targeting molecules. Ma and colleagues encapsulate CuO NPs in erythrocyte membranes incubated with a construct made from peptides, PEG, and phospholipid. The assembly was shown to have SOD-like activity and H_2_O_2_ and superoxide removal capacity in a 3xTg-AD mouse model (Ma et al., 2020; Figure 1C). Yu et al. (2020) also used the 3xTg-AD mouse model to test metal oxide frameworks to relieve AD-related oxidative stress. Yu took cerium oxide NPs and retinoic acid (RA) and enclosed them in MIL-100 (Fe) frameworks made from iron and trimesic acid. The framework preferentially breaks down in inflammatory environments with H_2_O_2_ allowing RA to upregulate neurogenesis genes while the ceria particles act as H_2_O_2_ antioxidants (Yu et al., 2020; Figure 1F). Carbon-based nanomaterials hold some promise for use as antioxidants, neuroprotectants and radical species scavengers. Like NPs, carbon based nano assemblies can pass through the BBB. These include carbon nanotubes, PEG functionalized carbon clusters, and fullerenes (Eleftheriadou et al., 2020). For example, Dal Bosco found PEGylated single walled carbon nanotubes induce a delayed antioxidant response in rat hippocampus without any lasting effects on memory or locomotion (Dal Bosco et al., 2015). However, some CNT formulations have been found to have opposite effects depending on their structural features, such as ROS formation or antioxidant depletion (van Berlo et al., 2012).

### Antioxidant Drug Delivery Nanomaterials

In this section, we focus on nanomaterials that are indirectly targeting oxidative stress and mitochondrial damage by the delivery of antioxidants. Materials discussed include NPs, nanoemulsions, liposomes, and exosomes. The materials improve localized delivery to the brain through better BBB penetration, functionalization to better target α-syn, or by escaping immune detection.

Nanoparticles (NPs) have potential for use as effective carriers of therapeutics to improve BBB penetration. The potential of NPs to increase the efficacy of PD therapies is exemplified by the case of lipoic acid, a molecule naturally present in the mitochondria with powerful anti-inflammatory and antioxidant properties capable of attenuating oxidative stress (Moura et al., 2015; Molz and Schröder, 2017; Andreeva-Gateva et al., 2020). The administration of lipoic acid is challenging due to the molecule’s short-half life and limited bioavailability caused by hepatic degradation (Teichert et al., 2003; Salehi et al., 2019). NPs can enhance the yield of intracellular lipoic acid delivery. Piersimoni et al. found that lipoic acid capped gold NPs (GNPs-LA) were biocompatible, capable of easily entering cells, and increased the efficacy of drug-delivery *in vitro*. GNPs-LA restored intracellular physiological conditions by preventing ROS formation and restoring normal mitochondrial function (Piersimoni et al., 2020). However, further study is still necessary to elucidate the exact molecular mechanism behind the GNPs-LA system.

In addition to NPs, nanoemulsions are a colloidal particulate system that can improve delivery across the BBB through noninvasive and direct delivery of lipophilic drug either through the mucosa intranasally or by improving the solubility and oral bioavailability of lipophilic drugs. Antioxidants, vitamin E, and coenzyme Q10, have both been incorporated in nanoemulsions for intranasal and oral delivery, respectively (Gupta et al., 2018; Gaba et al., 2019). Both were shown to improve behavior effects and reduce oxidative stress in PD rat models, with no signs of ciliotoxicity.

Additional carriers for antioxidants include nanoliposomes and exosomes, which can enhance BBB penetration. Antioxidants resveratrol (RES), baicalein, and epigallocatechin gallate (EGCG) have additionally been found to inhibit α-syn aggregation (Stojanoviæ et al., 2001; Wu et al., 2011; Xu et al., 2016; Aliakbari et al., 2018). Resveratrol-loaded liposomes performed better than free RES in protection from oxidative stress in rat models, and similarly, baicalein-loaded nanoliposomes exhibited increased delivery, stability, and internalization of baicalein *in vitro*. Liposomes have also been used to deliver drugs in combination, including curcumin paired with RES and EGCG paired with RES (Huang et al., 2019; Kuo et al., 2021). Functionalization of the RES-EGCG loaded liposomes with leptin and 1-palmitoyl-2-oleoyl-sn-glycero-3-phosphate (PA) increased BBB penetration and α-syn targeting through binding to the leptin receptor and PA binding to α-syn, with increased delivery, reduced apoptosis and oxidative stress, and neuronal rescue (Kuo et al., 2021). Exosomes additionally can be embedded with adhesive proteins and avoid phagocytosis by the immune system. An antioxidant catalase loaded exosome was developed by Haney et al. (2015), which resulted in high loading efficiency, sustained release, and catalase preservation against protease degradation. These exosomes were readily taken up by neuronal cells *in vitro* and were substantially detected in PD mouse brains after intranasal administration with significant neuroprotective effects (Haney et al., 2015).

There have been recent studies that use hydrogel-nanomaterial composites or nanogels to improve antioxidant drug delivery in AD. Rajput et al. (2018) developed xanthan and gellan gum gels containing nanostructured lipid formations to solubilize RES for treatment of AD using a nasal route. In the work of Elnaggar et al., chitosan nanogels were shown to aid in the delivery of a neuroprotectant used to treat AD, piperine, intranasally. The chitosan gels have mucoadhesive properties which allowed effective, non-invasive piperine delivery with a fraction of the oral dose in AD rat models (Elnaggar et al., 2015). Cardia and colleagues use chitosan hydrogel NPs to improve the delivery of neuroprotective progesterone and found increased progesterone concentration in rats that took the hydrogels intranasally (Lai et al., 2019).

Enhanced nasal delivery could allow the use of normally minimally bioavailable anti-AD drugs such as Timosaponin BII. Chen administered timosaponin BII to mice intranasally in a formulation of in-situ gellan gum hydrogels (Chen W. et al., 2020). Salatin et al. use a surfactant (F-127) hydrogel to embed NPs made from Eudragit RL-100 polymer. The hydrogel acts as a thermoreversible delivery system that enables controlled, intranasal delivery of the AD drug, Rivastigmine (Salatin et al., 2020).

## Nanotechnology Approaches Targeting Fibrillization Processes

### Nanomaterials Approaches Targeting α-Synuclein Aggregation in PD

Strategies to decrease α-syn aggregation involve targeting the misfolded α-syn fibrils themselves or preventing increased expression of them. Here we discuss graphene quantum dots and cerium oxide NPs, which bind to α-syn and disaggregate fibrils directly, as well as a modified exosome that targets the central nervous system to deliver α-syn siRNA and prevent α-syn translation.

Graphene quantum dots (GQDs) are NPs composed of layers of graphene and are approximately 100 nm in size. Kim et al. (2018) found that GQDs successfully pass through the BBB and bind to α-syn fibrils, a process which inhibits α-syn fibrillization and disaggregates fibrillated α-syn in a time-dependent manner. GQDs also inhibited transmission of α-syn PFFs. Kim et al. additionally demonstrated that GQDs display neuroprotective properties. After treatment of α-syn PFFs induced synaptic dysfunction and mitochondrial damage, GQDs were found to restore reduced synaptic protein levels, relieve the effects of α-syn-induced mitochondrial damage, and reduce the formation of Lewy body/neurites. Additionally, GQDs did not cause any significant long-term toxicity *in vitro* or *in vivo* (Kim et al., 2018). Although studies have demonstrated the protective effect of GQDs, further studies are needed to understand the mechanisms.

Additionally, the cerium oxide NPs, as previously discussed in section 2.1, have been shown to fit best into the active site of α-syn and disaggregate fibrillar α-syn *in vivo* compared to other biomaterials such as gold and superparamagnetic iron-oxide NPs through recent molecular docking studies (Kaushik et al., 2018; Zand et al., 2019). In Ruotolo’s study, CeO_2_ NP treatment on a yeast model with cells overexpressing human α-syn significantly reduced α-syn cytotoxicity in a dose-dependent manner through inhibition of α-syn cytoplasmic inclusion formation and decreased mitochondrial damage as discussed (Ruotolo et al., 2020).

Altering the gene expression of α-syn is an additional approach to decreasing misfolded α-syn levels. Exosomes can be modified to enhance delivery as Cooper et al. did with the central nervous system-specific rabies virus glycoprotein peptide (RVG) to deliver siRNA to reduce α-syn expression. The modified RVG-exosome has therapeutic potential in delivery of α-syn siRNA to delay and reverse alpha synucleinopathies (Cooper et al., 2014).

### Nanomaterials Approaches Targeting Aβ and Tau Aggregation in AD

In older studies, water soluble gold NPs (AuNP) synthesized in the presence of sodium citrate and functionalized with a peptide to form AuNP-Cys-Leu-Pro-Phe-Phe-AspNH_2_ were found to block amyloid fibril growth in a gel media when irradiated in a magnetic field. When the surfaces of the gold particles were heated using inductive coupling in a magnetic field, they transmitted their heat to surrounding tissues, consequently disrupting amyloid deposits (Kogan et al., 2006).

B -casein (βCas) proteins have chaperone-like activity, stemming from their lack of tertiary structure, existence as heterogeneous oligomers, and ability to bind to a range of partially folded proteins thus preventing their aggregation (Thorn et al., 2009). Coating βCas with AuNPs and delivering them intracardially has been found to control the toxicity of Aβ_42_ when induced in the brain of zebrafish larvae and adults. βCas alone does not exhibit any controlling activity on Aβ_42_ activity, suggesting that AuNPs are critical in delivering βCas to Aβ_42_ aggregate regions in the brain (Javed et al., 2019). AuNPs serve as the foundation for the design of a polyoxometalate-based nanozyme with a multifaceted approach to AD mitigation (Gao et al., 2016). Gao and colleagues functionalized AuNPs with a serine protease-like complex consisting of polyoxometalate conjugated to an octapeptide motif which was found to simultaneously target Aβ fibrilization, ROS, and metal ion accumulation through protease-like activity, Cu scavenging, and metal chelation, respectively (2016).

The CuO-based nanozymes of Ma and colleagues were functionalized with a KLVFF-modified PEG with phospholipid to embed the construct into erythrocyte membrane. The KLVFF motif binds to Aβ in blood circulation and the biomimetic construct was found to improve peripheral Aβ clearance when injected into mice (Ma et al., 2020). Recent cellular and mouse model study shows some promise that selenium-chondroitin sulfate NPs could be a multifunctional agent for AD treatment, inhibiting both Aβ aggregation and attenuating the hyperphosphorylation of tau at Ser396 and Ser404 by regulating glycogen synthase kinase 3 β (Guo et al., 2016; He et al., 2018).

Protein-capped (PC) metal NPs were synthesized by Sonawane et al. (2019) by exposing the fungal species *F. oxysporum* and *Verticillium* spores to various metal solutions to create different types of NPs. Aqueous mixtures of ferricyanide or ferrocyanide were used to create magnetite NPs, and Cd^2+^ and SO_4_^2–^ were used to create CdS NPs. PC-metal NPs inhibit tau fibrillization by reducing hyperphosphorylation. Sonawane et al.’s study showed the direct effect of PC-metal NPs on tau aggregates, as they were quantifiably inhibited, and mature fibrils readily dissolved. Uncapped CdS NPs are toxic to bacterial and HeLa cells, due to oxidative stress caused by increased concentrations of reactive oxygen species (Hossain and Mukherjee, 2013). However, capping the NP makes them far more biocompatible and represents a viable therapeutic route.

Vakilinezhad et al. (2018) found that nanoformulations of histone deacetylase (HDAC) inhibitor, nicotinamide also inhibits tau hyperphosphorylation. While large doses of nicotinamide may be hepatotoxic (Knip et al., 2000), the researchers used solid lipid NPs (SLN) to provide localized, controlled release of nicotinamide by making them in different sizes. The SLNs were made from the physiological lipids, phosphatidylserine, and phosphatidic acid. Inhibition of hyperphosphorylation of tau in a rat model was confirmed by an enzyme-linked immunosorbent assay, making it a possible candidate for AD treatment.

Photosensitizing materials are a recent area of study in the development of anti-Aβ therapies. These materials can have high affinity for Aβ and generate oxidative stress in response to light, thereby impeding Aβ aggregates (Hirabayashi et al., 2014; Mangione et al., 2015). Several nanoassemblies are being developed as light-responsive anti-Aβ agents. Fullerene is known to block Aβ aggregation. Tanimoto et al. (2012) hybridized fullerene with hydrophilic moieties to increase solubility while lowering cytotoxicity. When exposed to UV, the fullerene denatures Aβ_42_ monomers and oligomers (Tanimoto et al., 2012). Chung et al. (2017) used 10 nm, carbon nanodots functionalized with polyethyleneimine to inhibit the aggregation of Aβ_42_ using only visible light. Graphitic carbon nitride nanosheets have been used with visible light to suppress amyloidosis through the generation of reactive oxygen species from photochemical reactions. These reactive oxygen species then oxidize Aβ_42_ (Chung et al., 2016). Zhang and colleagues use photodynamic micelles functionalized with light-sensitive chlorin e6 to inhibit and degrade Aβ in 655 nm light (Zhang et al., 2016). In recent work, iron copper selenide NPs have had success preventing amyloidopathy in mouse models with near-infrared illumination (Zhang H. et al., 2020).

Common hydrogels made from collagen, agarose, HA, and PEG were all found to have neuroprotective effects in the presence of Aβ. However, Simpson et al. (2020) found that non-functionalized hydrogels thermodynamically favor Aβ aggregation due to confinement effects. In the work of Jiang, hydrogels made from conjugated hyaluronic acid (HA) and curcumin were observed to slow down Aβ aggregation through opposing forces caused by its hydrophobic binding of curcumin and electrostatic repulsion of HA (Jiang et al., 2016, 2018). Yau and colleagues seeded Aβ fibril fragments in *N*,*N*’−methylene−*bis*−acrylamide and *bis*−acryloylcystamine hydrogels and found it successfully sequestrated excess Aβ in solution (Yau and Tycko, 2018).

## Nanotechnology Targeting Inflammation

Anti-inflammatory drugs have been used to treat both PD and AD, but nanoemulsions and NPs can improve delivery through direct delivery from nasal mucosa or improved BBB penetrance.

Lipophilic anti-inflammatory medication, ibuprofen, has neuroprotective effects, and to improve its delivery to the brain, Mandal et al. (2016) loaded ibuprofen into sodium hyaluronate based mucoadhesive nanoemulsion (MNEI). This intranasal nanoemulsion can more directly deliver ibuprofen to the brain through nasal mucosa than traditional oral routes. 1-methyl-4-phenyl-1,2,3,6-tetrahydropyridine mice models were treated with ibuprofen for two weeks as an intranasal plain drug solution and with the nanoemulsion. Nanoemulsion delivery of ibuprofen significantly reduced MPTP-mediated dopamine depletion (Mandal et al., 2016).

For the treatment of AD, NPs of anti-inflammatory molecule quercetin encapsulated in β-cyclodextrin-dodecylcarbonate were shown to have increased anti-inflammatory effects compared to free quercetin *in vitro* with the goal of improving permeation and bioavailability (Testa et al., 2014). Curcumin loaded solid lipid NPs showed improved bioavailability, a greater inhibition of NO production and reduced inflammatory markers compared to conventional curcumin in a dose-dependent manner *in vitro* (Ganesan et al., 2019). A modified Poly(lactic-co-glycolic acid) (PLGA) NP was conjugated with CD47 extracellular domain via ROS-responsive phenylborate ester bond, which acts as a “do not eat me” signal, and a BBB penetrating peptide (CRT) encapsulating a microglia modulation agent necrostatin-1 (Nec-1). The NP efficiently increased the half-life of Nec-1 via prevention of phagocyte engulfment as well as increased brain distribution. The encapsulated Nec-1s are released as the ROS-sensitive bond between CD47 and the NPs are broken in AD mouse brains, allowing microglia engulfment of Nec-1 to modulate pathogenic microglia, reducing neuroinflammation (Zhang L. et al., 2020). Additionally, mitochondria-targeted quantum dot nanozymes were designed to switch microglia from proinflammatory M1 phenotype to the anti-inflammatory M2 phenotype, which can mitigate Aβ aggregate-mediated neurotoxicity. These nanozymes effectively crossed the BBB, escaped from lysosomes, targeted the mitochondria, and prevented spontaneous neuroinflammation through regulation of proinflammatory mediators *in vitro* and in AD mouse models (Ren et al., 2020).

## Nanotechnology for Regenerative Medicine

Currently, there is no cure for PD or AD, but efforts in regenerative medicine are aimed at promoting neuronal growth and axonal extension to repair cell damage of both diseases. To stimulate guidance of axonal growth and cellular survival, neurotrophic factors can be delivered via nanomaterials to extend their half-life and enhance delivery to the brain. Additionally, scaffolds not only provide a vehicle to deliver neurotrophic factors but are also an ideal physical structure with optimal microenvironment to promote cell growth of host neurons or implanted neurons.

Glial cell derived neurotrophic factor (GDNF) has a short *in vivo* half-life, but delivery in PLGA microparticles increased half-life, improved motor function and dopaminergic neuron restoration, with no adverse effects on immunogenicity, cerebellar degeneration, or weight loss *in vivo* (Garbayo et al., 2016). GDNFs delivered in hydrogel scaffolds have additional physical support and promote cell survival and axonal growth *in vivo* and *in vitro* (Wang et al., 2016; Ucar and Humpel, 2019). In addition to collagen hydrogel scaffolds, cryogels and microcontact printing also showed enhancement of axonal fiber growth *in vitro* (Ucar et al., 2021). As hydrogel scaffolds provide favorable environments for neuronal growth and protection from host immune responses, the encapsulation of dopaminergic neurons in scaffolds for transplantation present improved graft survival in PD. The hydrogel can be functionalized with RGD cell adhesion peptide and heparin to secure cells and immobilize growth factors for *in vitro* maturation of neurons (Adil et al., 2017). Hydrogel scaffolds can additionally be structured to mimic the nigrostriatal pathway architecture with micro-columns of dopaminergic neurons as in the work of Struzyna et al. (2018).

Self-assembling peptides have the potential to improve the benefits of neural stem cell (NSC) transplantation to treat AD (Bordoni et al., 2020). Alternating amino acid sequences can be designed to create a nanofibril scaffold that can support neuronal growth and differentiation. Guo-hong Cui and colleagues designed a self-assembling peptide to successfully treat AD in a mouse model. Cui observed improved NSC survival, improved NSC differentiation, and lowered Aβ levels using peptides modeled after laminin (Cui et al., 2016). Yu et al. (2014) used lactoferrin peptides in a polymersome (PEG-PLGA) assembly to enhance delivery of humanin, a peptide known to inhibit AD-related cell death caused by Aβ.

Dongqin Yu used the previously mentioned metal oxide frameworked nanozymes functionalized with siRNA to downregulate SOX9, known to cause gliogenesis (2020). At the same time, RA within the framework was released to upregulate neuronal genes, creating a vital balance for neurogenesis without excessive glial response (Yu et al., 2020).

## Conclusion and Future Perspectives

Amid contemporary challenges in targeted drug delivery and barriers to transport of drugs across the BBB, nanomaterials are emerging as promising approaches for the treatment of neurodegenerative disorders. This review provides insights into the role nanomaterials can play in improving the delivery of therapeutics to patients of PD and AD, the two most common neurodegenerative diseases, by targeting the pathological mechanisms of oxidative stress or mitochondrial damage, protein fibrillization, inflammation, and cell death (Table 1).

Current and emerging challenges in NP research include concerns regarding the toxicity of NPs. Research has shown that the very properties that account for the benefits of NPs may also contribute to toxic effects (Aillon et al., 2009; Song et al., 2016; Mohammadi and Nikkhah, 2017). Generally, neurotoxicity from NPs stems from their production of reactive oxygen species, causing oxidative stress (Teleanu et al., 2018). However, some NP biocompatibility improves with certain modifications. Looking forward, NP toxicity must be researched and thoroughly addressed, and efforts must be made to reduce or eliminate any toxic effects during the development phase.

The effect of NPs on pathological protein aggregation are found to vary depending on their composition, size, shape, and charge. Further study is necessary in identifying how NPs act under various combinations of these factors. *In vitro* and *in vivo* studies are both crucial to the study and identification of the best NP-based treatment for neurodegenerative diseases.

Additionally, the recent innovations in nanozymes present exciting new possibilities of addressing oxidative stress and inflammation in AD and PD, but more work needs to be done for safe clinical use. Further research on the catalytic mechanisms of nanozymes will be necessary to have the understanding to optimize the structure, function, and regulation of catalytic activity. Additionally, the biocompatibility and nano-bio interactions of nanozymes should be further explored to ensure safety and efficacy of nanozyme treatment (Wang et al., 2018; Tian et al., 2020).

The modifiability and freedom in design of various nanomaterials present a wide variety of delivery strategies for medications by enhancing transport across the BBB and enabling targeted delivery or additional functions while accommodating drug chemistries and solubilities and avoiding immune system detection. In regenerative medicine, which aims to reverse the damage of neurodegeneration, materials, such as hydrogel scaffolds, could play a crucial role in creating an optimal physical and microenvironment to support neuronal growth and axonal extension. However, the most ideal microenvironments and drug combinations must be better understood.

Nanomaterials represent an ever-growing field to approach neurodegenerative disease treatment, both in transforming the delivery of therapeutic agents and in creating an entirely new class of therapies targeting canonically challenging disorders.

## Author Contributions

MP and SK completed initial nanoparticle literature review for AD. SP completed the initial nanoparticle literature review for PD. AL completed nanomaterial literature review outside of nanoparticles for PD. JT completed nanomaterial literature review for AD. XM and WH supervised the manuscript preparation and writing. All authors contributed to the article and approved the submitted version.

## Conflict of Interest

The authors declare that the research was conducted in the absence of any commercial or financial relationships that could be construed as a potential conflict of interest.
